# Exploring the anti-obesity effects of kimchi through enhanced thermogenesis in differentiated T37i brown adipocytes

**DOI:** 10.29219/fnr.v68.10738

**Published:** 2024-08-29

**Authors:** Ye-Rang Yun, Ji-Eun Lee, Seongsoo Lee, Sung Wook Hong

**Affiliations:** 1 World Institute of Kimchi, Gwangju, Republic of Korea; 2 Gwangju Center, Korea Basic Science Institute (KBSI), Gwangju, Republic of Korea

**Keywords:** kimchi, obesity, thermogenesis, brown adipocytes, gene expression

## Abstract

**Background:**

Previous research has demonstrated the anti-obesity effects of kimchi in 3T3-L1 adipocytes and mice with diet-induced obesity by assessing the expression of obesity-associated genes. Additionally, recent studies have identified mechanisms involving thermogenesis that support these effects.

**Objective:**

This study aims to further investigate the anti-obesity properties of kimchi, focusing on its impact on thermogenic activity in differentiated T37i brown adipocytes.

**Design:**

The study first evaluated the antioxidant potential of kimchi using total antioxidant capacity (TAC) and ferric reducing antioxidant power (FRAP) assays. Optimal differentiation conditions for T37i adipocytes were established before proceeding with evaluations of cell viability, intracellular triglyceride (TG) content, lipid accumulation, and the expression of genes and proteins related to obesity and thermogenesis.

**Results:**

Kimchi maintained over 90% cell viability in T37i adipocytes at concentrations up to 1,000 μg/mL. Efficient differentiation of T37i preadipocytes was achieved using a medium containing 10% calf serum, 2 nM 3,3’,5-triiodo-L-thyronin (T3), and 100 nM insulin. Kimchi significantly reduced intracellular TG levels and lipid accumulation, compared to the control group, and enhanced the expression of genes and proteins related to thermogenesis while reducing the expression of obesity-related genes.

**Discussion:**

The findings suggest that kimchi exerts its anti-obesity effects by modulating thermogenic and obesity-related pathways in brown adipocytes, which may be partially attributed to its antioxidant properties.

**Conclusions:**

Kimchi shows promise as a preventive measure against obesity by influencing metabolic pathways associated with both obesity and thermogenesis in T37i brown adipocytes.

## Graphic Abstract

**Figure:**
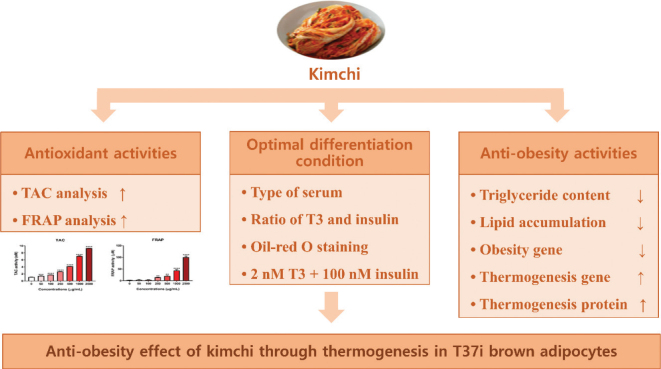

## Popular scientific summary

- We establish the more efficient differentiation condition of T37i brown adipocyte.
- Kimchi inhibits intracellular triglyceride and lipid accumulation in T37i brown adipocytes.
- Kimchi shows an anti-obesity effect by regulation of obesity-related biomarkers as well as thermogenesis-related biomarkers.
- This study is the first evidence of kimchi’s anti-obesity impact through the regulation of thermogenic effect.

Interest in kimchi has been steadily increasing because of its recognition as a health beneficial food, particularly in the context of the COVID-19 pandemic. Consequently, numerous studies have delved into its health advantages (1–3). Kim et al. demonstrated that *Lactobacillus sakei* HEM 224, extracted from kimchi, alleviated inflammatory conditions in the gastrointestinal and respiratory tracts by bolstering the epithelial barrier and modulating the immune response (3). In another investigation, kimchi consumption effectively mitigated obesity by regulating neuroinflammation and addressing microbiota imbalance (1). The health-promoting properties of kimchi have been attributed to its active constituents, namely lactic acid bacteria (LAB), and the metabolites resulting from kimchi fermentation (4, 5). Our prior studies have shown that these active constituents can inhibit endoplasmic reticulum (ER) stress-induced hepatic steatosis both *in vitro* and *in vivo* (4). Furthermore, kimchi supplemented with LAB, known for their anti-obesity effects, exhibited enhanced efficacy against obesity in 3T3-L1 cells (5).

Among kimchi’s various health benefits, its anti-obesity effects have been extensively studied. Previous investigations have focused on the anti-obesity properties of kimchi using 3T3-L1 white adipocytes and high-fat diet-fed animal models (5–7). Our previous research demonstrated the anti-obesity potential of LAB-containing kimchi in differentiated 3T3-L1 cells (5). Hong et al. documented that kimchi enriched with catechins and LAB exhibited anti-obesity effects in high-fat diet-induced obese mice by modulating lipid metabolism and the gut microbiome (6). Similarly, our earlier study revealed that kimchi supplemented with citrus concentrate exerted anti-obesity effects in high-fat diet-induced obese mice (7). Additionally, we confirmed the anti-obesity effects of LAB-fortified kimchi in high-fat diet-induced obese mice (8).

Recent studies have explored the anti-obesity effects of thermogenesis by assessing the expression of thermogenic-specific genes and proteins, such as uncoupling protein-1 (UCP-1), peroxisome proliferator-activated receptor gamma coactivator 1-alpha (PGC-1α), and PR domain-containing 16 (PRDM16) in white adipocytes and tissues, aligning with current research trends (9, 10). Choi et al. observed that treatment with *Paeonia lactiflora* root enhanced levels of UCP-1, PGC-1α, and PRDM16 in 3T3-L1 cells, thereby exerting anti-obesity effects (9). Similarly, in obese mice, (20R)-panaxadiol demonstrated anti-obesity effects by upregulating the expression of thermogenic proteins (10). Among these anti-obesity mechanisms through thermogenesis, AMP-activated protein kinase (AMPK) pathway is a predominant pathway that is closely involved in brown adipose tissue differentiation and activation (11). AMPK activation not only indirectly modulated a UCP-1 protein, which increase thermogenic activity, but also induced related biomarker expression (12). Similarly, kimchi active components and kimchi also induced AMPK activation in our previous studies (13, 14). Therefore, kimchi anti-obesity effect of kimchi through thermogenesis is expected. Although studies on the anti-obesity effect of thermogenesis have been extensively conducted, there is little study focusing on brown adipocytes.

Thus, this study aimed to explore the anti-obesity effects of thermogenesis in T37i brown adipocytes. Initially, we assessed the antioxidant activity of kimchi. Subsequently, after establishing optimal differentiation conditions for T37i adipocytes, we investigated the anti-obesity properties of kimchi by evaluating parameters such as cell viability, triglyceride (TG) levels, lipid accumulation, and the expression of obesity/thermogenesis-related genes and proteins.

## Materials and methods

### Chemicals

3,3’,5-Triiodo-L-thyronin (T3), insulin, oil-red O (ORO) solution, and a total antioxidant capacity (TAC) kit were sourced from Sigma-Aldrich (MO, USA). The FRAP assay kit was acquired from Ann Arbor Biotechnology (MI, USA). The Cell Counting Kit-8 (CCK-8) was obtained from Dojindo (Kumamoto, Japan). The triglyceride assay kit was procured from Asan Pharmaceutical (Seoul, Korea). The TRIzol^TM^ reagent was purchased from Invitrogen (Carlsbad, CA, USA). The TOPScript^TM^ cDNA Synthesis Kit and SYBR Green premix were obtained from Enzynomics, Inc. (Daejeon, Korea). RIPA buffer was sourced from Thermo Fisher Scientific (Waltham, MA, USA). Primary and secondary antibodies for western blotting were purchased from Cell Signaling Technology (Danvers, MA, USA).

### Preparation of kimchi

Kimchi was prepared using brined cabbage, red pepper, garlic, ginger, onion, radish, and other ingredients. Fermentation of kimchi was carried out until reaching a pH of 4.2 at 6°C, followed by freeze-drying for subsequent analysis.

### Capsaicinoid analysis

For capsaicinoid extraction, 2.5 g of homogenized kimchi was weighed and combined with glass beads (size 4) in 22 mL clear vials equipped with a PTFE liner (Supelco, USA). Subsequently, 15 mL of methanol was added. The vials were capped, heated in a block at 90°C for 1 h, cooled to room temperature (RT, 15–25°C), transferred into 25 mL volumetric flasks, and filled with methanol. Samples were then filtered through disposable syringes with 0.2 μm filters (Millipore, USA) and subjected to capsaicinoid content determination using high-performance liquid chromatography (HPLC) with a Lachrom Ultra C18 column (50 mm × 2.0 mm, 2 μm; Hitachi, Japan) and an Agilent 1260 infinity LC system equipped with a fluorescence detector (Agilent Technologies, USA). The analysis conditions were as follows: mobile phase A, 0.1% acetic acid; mobile phase B, acetonitrile; isocratic condition A: *B* = 6:4; flow rate of 0.6 mL/min; excitation wavelength of 280 nm, emission wavelength of 325 nm; column temperature of 25°C; and injection volume of 2 μL.

### Antioxidant activity analysis

The antioxidant activity of kimchi was assessed through TAC and FRAP assays. TAC analysis was conducted using a commercial kit. Cu^2+^ working solution was incubated with various concentrations of kimchi samples (0, 50, 100, 250, 500, 1,000, and 2,500 μg/mL) for 90 min at RT. Absorbance was measured at 570 nm, and the TAC value was expressed in nM based on the Trolox standard. FRAP analysis was performed using a commercial kit. The FRAP color solution was incubated with various concentrations of kimchi samples (0, 50, 100, 250, 500, 1,000, and 2,500 μg/mL) for 30 min at RT, and absorbance was measured at 560 nm. FRAP values were expressed in μM based on the Fe (II) standard.

### Cell culture and cell viability analysis

T37i mouse brown adipocytes were procured from Millipore (Billerica, MA, USA) and cultured in DMEM/F12 supplemented with HEPES, l-glutamine (10% fetal bovine serum (FBS), and 1% penicillin/streptomycin). Cell viability was assessed using a CCK-8 kit. T37i preadipocytes (1 × 10^4^ cells/well) were seeded and treated with kimchi (0, 50, 100, 250, 500, 1,000, and 2,500 μg/mL) for 1 day. After washing with PBS, cells were incubated with 20 μL CCK-8 solution for 2 h, and absorbance was measured at 450 nm.

### Optimal differentiation condition analysis

To determine the optimal differentiation conditions for T37i preadipocytes, a differentiation condition test was conducted considering the type of serum (FBS and calf serum (CS)) and the composition of the differentiation medium (ratio of T3 to insulin: 2 nM + 20 nM, 2 nM + 40 nM, and 2 nM + 100 nM). Optimal conditions were evaluated based on ORO-stained images and intensities. Subsequent experiments were performed under the identified optimal conditions (2 nM T3 and 100 nM insulin for 9 days).

### Intracellular triglyceride content analysis

Lipids from differentiated T37i adipocytes were extracted using a solvent mixture (700 μL, chloroform/methanol/H_2_O mixture, 8:4:3, v/v/v), as previously described (15). Extracted lipids were incubated at room temperature for 60 min, and the organic layer was obtained by centrifugation at 800 × *g* for 10 min and dried. Dried lipids were dissolved in ethanol (20 μL), and triglyceride content was measured (Asan Pharmaceutical, Seoul, Korea).

### Oil-red O staining analysis

Differentiated T37i adipocytes were rinsed and fixed in 10% formalin for 30 min. Subsequently, the cells were stained with ORO solution for 15 min at RT and then washed. For lipid quantification, the stained cells were extracted with isopropanol, and the absorbance was measured at 510 nm.

### Quantitative real-time polymerase chain reaction analysis

Total RNA was extracted using the TRIzol^TM^ reagent, and cDNA synthesis was performed using a cDNA synthesis kit. Quantitative real-time polymerase chain reaction (qPCR) of the synthesized cDNA was performed using SYBR Green Premix and primers (Table 1). The qPCR protocol involved an initial activation step at 94°C for 10 min, followed by 45 cycles of denaturation at 94°C for 15 s, and annealing/extension at 60°C for 1 min. The qPCR results were normalized to those of *GAPDH*.

**Table 1:** Primer sequences for quantitative real-time PCR

Gene	Forward primer (5′-3′)	Reverse primer (5′-3′)
*GAPDH*	GTATGACTCCACTCACGGCAAA	GGTGTGGCTCCTGGAAGATG
Obesity-related gene		
*aP2*	CATGGCCAAGCCCAACAT	CGCCCAGTTTGAAGGAAATC
*C/EBPα*	AGGTGCTGGAGTTGACCAGT	CAGCCTAGAGATCCAGCGAC
*FAS*	TCTGAGCAGGTGCAGGAGGA	GTTGTTCCTCCAGTTCCGATTTGTA
*LXRα*	CTCAATGCCTGATGTTTCTCCT	TCCAACCCTATCCCTAAAGCAA
*PPARγ*	TGGAATTAGATGACAGCGACTTGG	CTGGAGCAGCTTGGCAAACA
*SREBP-1c*	AGAGGGTGAGCCTGACAA	CCTCTGCAATTTCCAGAT
Thermogenesis-related gene		
*CIDEA*	ATCACAACTGGCCTGGTTACG	TACTACCCGGTGTCCATTTCT
*CtBP1*	TTGGGCATCATTGGACTAGGT	TAACGCAGTCACTGTGGAAGA
*PGC-1α*	GCAGCCAAGACTCTGTATG	ATTGGTCGCTACACCACTTC
*PPIA*	CAAATGCTGGACCAAACACAA	GCCATCCAGCCATTCAGTCT
*PRDM16*	ATGCGAGGTCTGCCACAAGT	CTGCCAGGCGTGTAATGGTT
*UCP-1*	GGCCTCTACGACTCAGTCCA	TAAGCCGGCTGAGATCTTGT

PCR, Polymerase chain reaction.

### Western blot analysis

Differentiated T37i adipocytes were lysed with RIPA buffer (Invitrogen, Thermo Fisher Scientific, USA) on ice for 1 h, followed by centrifugation at 10,000 × *g* for 10 min at 4°C. Proteins (30 μg) were separated by electrophoresis and transferred to membranes (Bio-Rad, Hercules, CA, USA). The membranes were then incubated with primary antibodies, including GAPDH, UCP-1, PGC-1α, and PRDM16 (1:1,000, Cell Signaling Technology, Danvers, MA, USA), followed by incubation with anti-rabbit secondary antibodies (Cell Signaling Technology, Danvers, MA, USA). Protein bands were visualized using an enhanced chemiluminescence system (ECL Advance; GE Healthcare, Hatfield, UK). Protein density was quantified using the Image J 1.53 program (NIH, USA).

### Statistical analysis

All experiments were conducted in triplicate. Data are presented as mean ± standard deviation (SD). Significant differences were determined using Student’s *t*-test (two groups) and one-way analysis of variance (ANOVA, for three or more groups), followed by Dunnett’s multiple comparison test using GraphPad Prism 9 (GraphPad, Inc., San Diego, CA, USA). Statistical significance was set at *P* < 0.05.

## Results and discussion

### Capsaicinoid content of kimchi

**Fig. 1:**
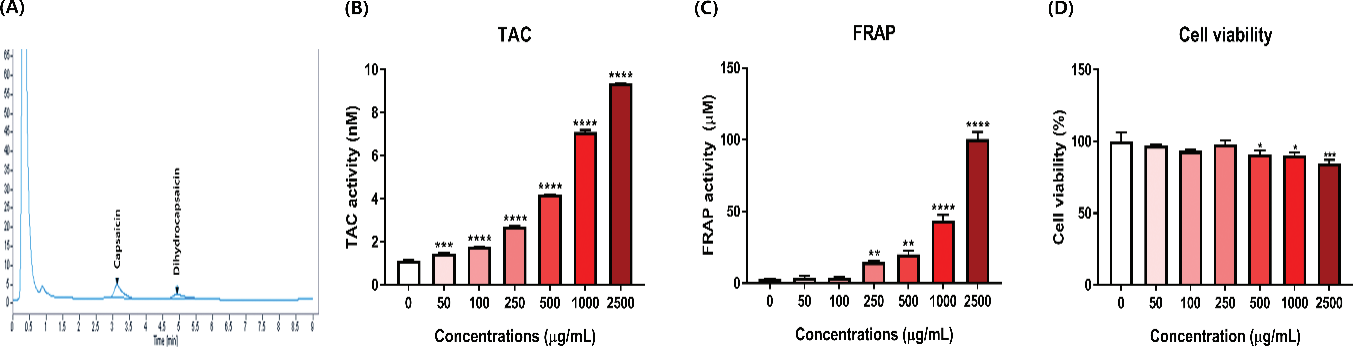
Capsaicin and dihydrocapsaicin contents and impact of kimchi on antioxidant activities and cell viability in T37i brown preadipocytes. (A) HPLC chromatograms of capsaicin and dihydrocapsaicin. (B) Total antioxidant capacity (TAC). (C) Ferric reducing antioxidant power (FRAP). (D) Cell viability. Results are presented as mean ± SD (n = 3). *P < 0.05, **P < 0.01, ***P < 0.001, and ****P < 0.0001, vs. non-treated group.

The capsaicinoid content was quantified using HPLC as a representative active constituent of kimchi. Capsaicin and dihydrocapsaicin were quantified by comparison with a reference standard. As depicted in Fig. 1A, capsaicin and dihydrocapsaicin contents were determined to be 96.31 mg/kg and 61.62 mg/kg, respectively. Kimchi was classified into five groups based on their capsaicin and dihydrocapsaicin contents, affecting perceived spiciness: control, mild, medium, hot and extreme kimchi according to Park’s classification (16). Our capsaicinoid content (157.93 mg/kg) aligned with the mild and medium levels, with 96.31 mg/kg for capsaicin and 61.62 mg/kg for dihydrocapsaicin.

### Antioxidant effect of kimchi

The antioxidant potential of kimchi was assessed using TAC and FRAP assays. Previous studies have consistently demonstrated the antioxidant activity of kimchi (15, 17). In line with these findings, both TAC and FRAP levels of kimchi exhibited a dose-dependent increase (Fig. 1B and 1C). Notably, the highest TAC and FRAP levels, at 2,500 μg/mL, were measured at 9.36 nM and 100.57 μM, respectively.

### Effect of kimchi on T37i preadipocyte viability

Figure 1D depicts the viability of T37i preadipocytes when treated with kimchi at various concentrations (0, 50, 100, 250, 500, 1,000, and 2,500 μg/mL). Preadipocyte viability remained above 90% up to a concentration of 1,000 μg/mL. Notably, the viability of kimchi-treated cells varied depending on the cell type and kimchi formulation. For instance, the viability of naturally fermented kimchi (NK) and starter kimchi (SK) was also approximately 80% up to 1,000 μg/mL; however, viability decreased to 60% at 2,500 μg/mL (5). Despite differences in kimchi formulations, cell viability remained above 80% up to 500 or 1,000 μg/mL (5, 7, 17). Based on these findings, subsequent experiments were conducted using 1,000 μg/mL of kimchi.

### Optimal differentiation conditions

**Fig. 2:**
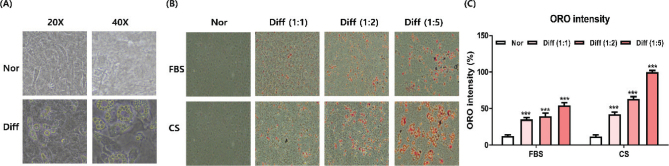
Optimization of differentiation conditions for T37i brown adipocytes. (A) Morphology of lipid droplets in differentiated T37i adipocytes. (B) Oil-red O (ORO) stained images. (C) ORO intensity. Results are presented as mean ± SD (*n* = 3). ***P < 0.001, vs. non-differentiated cells (Nor).

Brown adipocytes are characterized by a high mitochondrial density and small lipid droplet morphology. In this investigation, T37i brown adipocytes were primarily examined at 40× magnification, while 3T3-L1 white adipocytes were predominantly observed at 10× magnification (Fig. 2A). Previous research indicates that T37i adipocyte differentiation commonly occurs in differentiation media containing 2 nM T3 and 20 nM insulin for 6 days (18–20). However, well-differentiated T37i adipocytes were not observed in this study. To establish optimal differentiation conditions, experiments were conducted employing varying serum concentrations (FBS and calf serum) and ratios of T3 to insulin (1:1, 1:2, and 1:5), as depicted in Fig. 2B. Notably, the use of calf serum resulted in significantly higher differentiation of T37i adipocytes (*P* < 0.05, Fig. 2B and 2C). Moreover, higher concentrations of insulin led to significantly increased differentiation of T37i adipocytes (*P* < 0.05). Additionally, UCP-1 mRNA expression was confirmed in differentiated T37i adipocytes (data not shown). Based on these findings, differentiation experiments were conducted in DMEM/F12 supplemented with HEPES and l-glutamine, with 10% fetal calf serum containing 2 nM T3 and 100 nM insulin.

### Effect of kimchi on intracellular TG levels

**Fig. 3:**
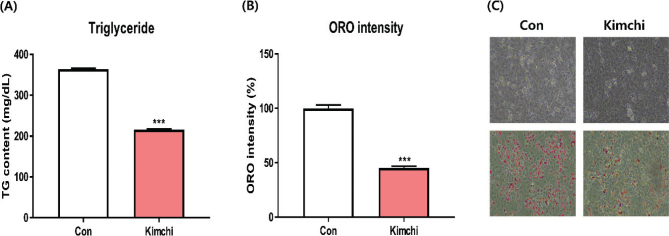
Influence of kimchi (1,000 μg/mL) on triglyceride (TG) content and lipid accumulation in differentiated T37i brown adipocytes. (A) TG content. (B) ORO intensity. (C) ORO-stained images. Results are presented as mean ± SD (*n* = 3). ***P < 0.001, vs. control group (Con).

In addition to UCP-1 expression, elevated TG levels are a characteristic feature of differentiated T37i adipocytes. The intracellular TG levels extracted from these differentiated adipocytes were assessed using a commercial kit. As depicted in Fig. 3A, the intracellular TG level significantly increased with differentiation in the control group; however, kimchi significantly reduced intracellular TG levels (*P* < 0.05). According to Penfornis et al., the mineralocorticoid (MR) receptor markedly increases TG levels in a dose-dependent manner; nevertheless, MR antagonists RU-26752 and RU-38486 notably decrease TG levels in differentiated T37i adipocytes (19). Additionally, carbamazepine demonstrates a significant decrease in TG levels (21). Several studies have demonstrated that kimchi significantly reduces TG levels in differentiated 3T3-L1 adipocytes (5, 17, 22). This study represents the first documentation of kimchi’s suppressive effect on differentiated T37i adipocytes.

### Effect of kimchi on lipid accumulation

The impact of kimchi on lipid accumulation was assessed through ORO staining. As illustrated in Fig. 3B and 3C, ORO-stained lipid droplets were evident in the control (Con) group. However, kimchi significantly reduced the number of ORO-stained lipid droplets (*P* < 0.05). Consistent with the TG results, kimchi effectively curbed lipid accumulation in differentiated T37i adipocytes. Similar findings were observed in studies where the sex hormone progesterone reduced lipid accumulation by approximately 30% in ORO assays (23). Additionally, carbamazepine has been reported to inhibit lipid accumulation (21). Collectively, these results indicate that kimchi exerts anti-obesity effects by reducing intracellular TG levels and inhibiting lipid accumulation.

### Effect of kimchi on thermogenesis-related gene and protein levels

To corroborate the anti-obesity effects of kimchi, the expression of thermogenesis-related genes was evaluated. UCP-1, PGC-1α, and PRDM16 are well established as major contributors to thermogenesis, thus influencing anti-obesity effects (24). Consequently, their expression at both gene and protein levels has been widely investigated in various cellular and tissue contexts (25, 26). Moreover, additional genes such as cell death-inducing DNA fragmentation factor-like effector A (*CIDEA*), peptidylprolyl isomerase A (*PPIA*), and C-terminal binding protein 1 (*CtBP1*) have been identified as thermogenesis-related genes (27).

**Fig. 4:**
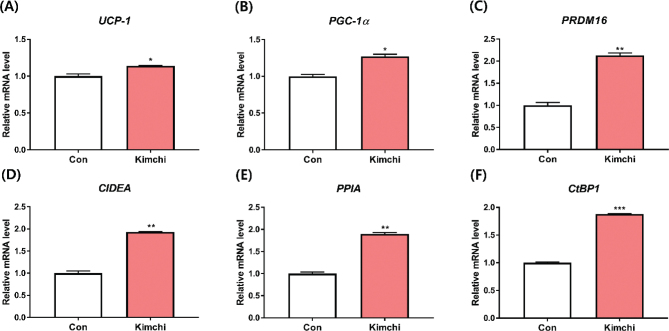
Effect of kimchi (1,000 μg/mL) on thermogenesis-related gene expression in differentiated T37i brown adipocytes. (A) *UCP-1*. (B) *PGC-1α*. (C) *PRDM16*. (D) *CIDEA*. (E) *PPIA*. (F) *CtBP1*. Results are presented as mean ± SD (*n* = 3). *P < 0.05, **P < 0.01, and ***P < 0.001, vs. control group (Con).

Fig. 4A–4F demonstrates a significant increase in the expression of thermogenesis-related genes following kimchi treatment compared to the control (*P* < 0.05). Consistent with these findings, visfatin has been reported to enhance the expression of thermogenic genes (28), whereas metformin has shown similar effects on thermogenic protein expression (29).

**Fig. 5:**
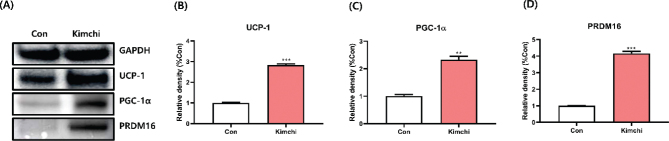
Effect of kimchi (1,000 μg/mL) on thermogenesis-related protein expression in differentiated T37i brown adipocytes. (A) Western blot images. (B) UCP-1 protein intensity. (C) PGC-1α protein intensity. (D) PRDM16 protein intensity. Results are presented as mean ± SD (*n* = 3). **P < 0.01 and ***P < 0.001, vs. control group (Con).

Consistent with the gene expression results, kimchi significantly upregulated the expression of key thermogenesis-related proteins, including UCP-1, PGC-1α, and PRDM16 (Fig. 5A–5D). Previous studies have consistently demonstrated that bioactive components enhance the expression of thermogenesis-related proteins. For example, ginsenosides Rb1 and Rg1, as well as carotene, have been shown to increase UCP-1, PGC-1α, and PRDM16 protein expression in 3T3-L1 cells (30, 31). These findings collectively underscore kimchi’s capacity to induce the expression of thermogenesis-related genes and proteins, thereby exerting an anti-obesity effect.

Although the mechanism study was not conduct in this study, our previous studies reported that kimchi active components and kimchi induced AMPK activation (13, 14). Therefore, these results suggest that kimchi shows the anti-obesity effect by thermogenesis in AMPK pathway. In the future, it is necessary to conduct and verify the mechanism in the AMPK pathway.

### Effect of kimchi on obesity-related gene expression

**Fig. 6:**
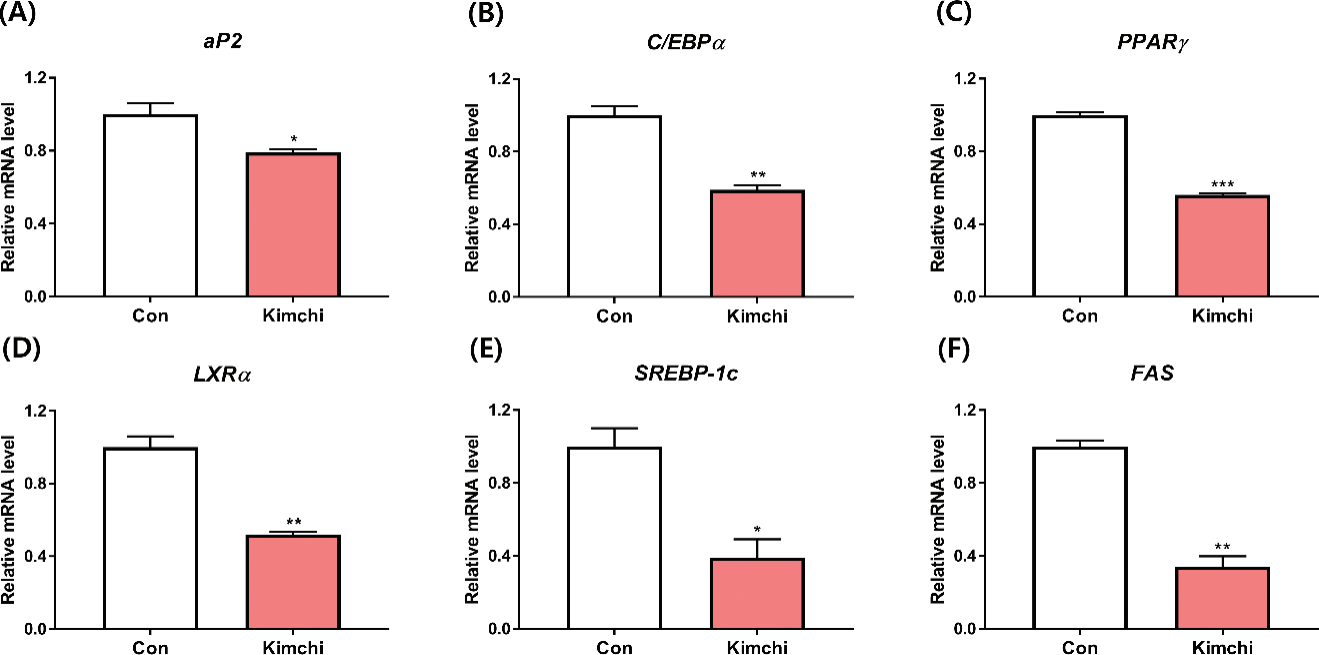
Effect of kimchi (1,000 μg/mL) on obesity-related gene expression in differentiated T37i brown adipocytes. (A) *aP2*. (B) *C/EBP*α. (C) *PPAR*γ. (D) *LXR*α. (E) *SREBP-1c*. (F) *FAS*. Results are presented as mean ± SD (*n* = 3). *P < 0.05, **P < 0.01, and ***P < 0.001, vs. control group (Con).

In addition to investigating the expression of genes related to thermogenesis, we examined the expression of genes associated with obesity. Kimchi notably downregulated the expression of obesity-related genes, including adipocyte fatty acid-binding protein (*aP2*), CCAAT/enhancer-binding protein alpha (*C/EBPα*), peroxisome proliferator-activated receptor gamma (*PPARγ*), liver X receptor alpha (*LXRα*), sterol regulatory element-binding protein-1c (*SREBP-1c*), and fatty acid synthase (*FAS*) (*P* < 0.05, as depicted in Fig. 6A–6F). Numerous studies have reported a significant reduction in the expression of obesity-related genes in differentiated 3T3-L1 adipocytes following kimchi treatment (32, 33).

These findings suggest that kimchi reduces the expression of thermogenesis-related genes and proteins, thereby eliciting an anti-obesity effect. In summary, the anti-obesity properties of kimchi were supported by the upregulation of thermogenic gene expression and the downregulation of obesity-related gene levels in differentiated T37i adipocytes.

## Conclusions

In this study, we assessed the antioxidant activity of kimchi and established optimal differentiation conditions for T37i brown adipocytes. By modulating the expression of genes and proteins related to obesity and thermogenesis, we induced anti-obesity effects. This study represents the first evidence of kimchi’s anti-obesity impact on differentiated T37i brown adipocytes through the regulation of obesity and thermogenesis pathways.

However, this study has some limitations. While we demonstrated the anti-obesity effects of kimchi on T37i adipocytes, further research is necessary to validate these effects on brown adipose tissue using animal models as well as mechanism study. Therefore, the verification of kimchi’s anti-obesity effects in mechanism and animal study is warranted.

## Data Availability

Not applicable.
